# Machinability of Rene 65 Superalloy

**DOI:** 10.3390/ma12122034

**Published:** 2019-06-25

**Authors:** Oluwole A. Olufayo, Hanqing Che, Victor Songmene, Christina Katsari, Stephen Yue

**Affiliations:** 1 Department of Mechanical Engineering, École de Technologie Supérieure, ÉTS, Montreal, QC H3C 1K3, Canada; oluwole-ayodeji.olufayo.1@ens.etsmtl.ca; 2 Department of Mining and Materials Engineering, McGill University, Montreal, QC H3A 0C5, Canada; hanqing.che@mail.mcgill.ca (H.C.); christina.katsari@mail.mcgill.ca (C.K.); steve.yue@mcgill.ca (S.Y.)

**Keywords:** Rene 65, nickel superalloy, drilling, cutting forces, surface finish, chip formation

## Abstract

Nickel-based superalloys are heavily used in the aerospace and power industries due to their excellent material and mechanical properties. They offer high strength at elevated temperatures, high hardness, corrosion resistance, thermal stability and improved fatigue properties. These superalloys were developed to address the demand for materials with the enhanced heat and stress capabilities needed to increase operational temperatures and speeds in jet and turbine engines. However, most of these properties come with machining difficulty, high wear rate, increased force and poor surface finish. Rene 65 is one of the next generation wrought nickel superalloys that addresses these demands at a reduced cost versus powder metallurgy superalloys. It is strengthened by the presence of gamma prime precipitates in its microstructure, which enhance its strength at high temperatures. Notwithstanding its advantages, Rene 65 must also deal with the reality of the poor workability and machinability generally associated with Ni-based superalloys. This study examines the machinability—using drilling tests—of Rene 65 and seeks to establish the influence of hardness (with varying microstructure) and cutting conditions on machinability indicators (surface finish, forces and chip formation). The experimental setup is based on a set of experimental drilling tests using three different heat-treated samples of varying hardness. The results indicate a negligible effect from material hardness, ranging from 41 HRC to 52 HRC, on generated cutting forces and a similarly low effect from cutting speeds. The feed rate was identified as the main factor of relevance in cutting force and chip morphology during the machining of this new superalloy.

## 1. Introduction

In recent years, the development and preparation of Ni-based wrought superalloys has become a global point of interest for research [1]. Technological innovations in alloy development and processing have led to the creation of high-strength Ni-based wrought superalloys. These materials are developed for aircraft turbines due to their elevated heat resistant properties. They possess similar or higher levels of creep strength as precision cast alloys used for gas turbine blades [2]. Their recent inclusion in aerospace has greatly contributed to gas turbine efficiency and thereby extended the life of turbines. Notwithstanding the high usage rate of these superalloys in industry, the machining of Ni-based superalloys is still a matter of great concern, as they are characterized by low workability and poor machinability. The workability of Rene 65 has been found to be acceptable when forging in the 1038 °C to 1079 °C temperature range [2] but its machinability is still an area that is yet to be addressed.

**Figure 1:**
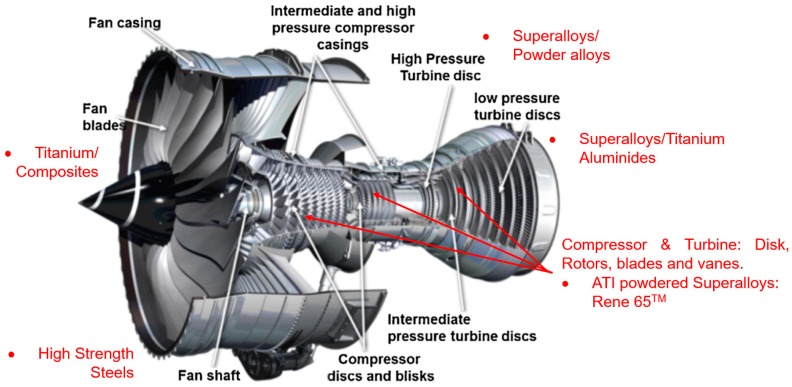
Some applications of Rene 65 in jet engine [4].

Rene 65, a newly developed wrought nickel superalloy, was designed to overcome the difficulties encountered in other Ni-base superalloys and can provide improved temperature capabilities (above 700 °C) relative to Inconel 718 and at a lower cost than powder metallurgy superalloys [3]. It is strengthened by the presence of gamma prime precipitates, which enhance its strength at elevated temperatures. Two phases can be found in the microstructure of the superalloys, namely, the γ and the γ′ phase [3]. Figure 1 shows the application of Rene 65 in a jet engine.

In the incoherent γ′ phase, superalloys lack the strengthening effects of the interface between γ′ and γ phases and show poor workability. However, this can be improved by applying heat treatment after hot forging to create a two-phase structure; furthermore, higher tensile strength has been associated with reduced workability in Ni superalloys [5]. 

**Figure 2:**
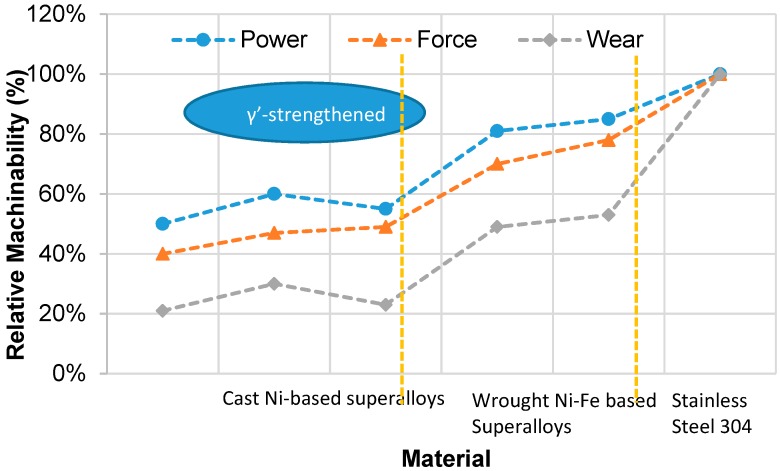
Machinability rankings for two classes of nickel-based superalloys relative to stainless steel 304 [10].

Machining is often used in the manufacture of superalloy parts. However, in the case of superalloy machining, the process is significantly more costly than for steels. This is due to limitations in cutting speeds when machining superalloys [6]. Despite recent advances in near-net shape operations such as precision casting and forging, machining still plays a crucial role in the finishing and rectification processes [7]. The term ‘machinability’ is used to estimate the degree of ease or difficulty by which a material can be machined. It represents this estimate based on indicators encountered in cutting operations and target conditions essential for part production. These include surface integrity, cutting forces, cutting temperature, tool wear, tool life, chip formation and burr size [8]. Materials exhibiting a higher machinability index are deemed to have good machinability. A study conducted by Andrew Henderson [9] identifies cast γ′-strengthened Ni-based superalloys as having a lower machinability than Ni-Fe superalloys. This machinability estimate was based on the cutting force, spindle power and tool wear (Figure 2).

During machining, poor thermal conductivity and work hardening lower the machinability index of superalloys. Machining superalloys also face a high wear rate, increased cutting force and reduced surface finish [7]. Additional challenges have been seen during drilling operations, ranging from the adverse effect of poor chip evacuation to an inefficient cooling practice [11]. 

To classify the machinability of this superalloy, an evaluation examining the process parameters of Rene 65 is conducted in this study to determine challenges faced during the machining of industrial jet engine parts. In the following sections, the properties impairing the machinability of Rene 65 will be discussed based on the following performance criteria: cutting force, surface roughness, chip formation and the influence of lubrication/coolant modes on tool condition.

### 1.1. Rene 65 and Other Ni-Base Superalloys

Nickel superalloys have at least a 50% wt nickel content, which acts as the main solution component. Nickel-based wrought superalloys also comprise the following elements: about 20% Cr, about 8% Al and Ti, 5–15% Co, and trace amounts of boron, zirconium, magnesium and carbon [12]. The chemical composition of several Ni-based superalloys is given in Table 1. Of these superalloys, the majority used in industry is the Inconel 718. AD730 and Rene 65 were developed to address the reduced strength of Inconel 718 at elevated temperatures. The workpiece used in this study is a Rene 65 Ni-based superalloy and it had the following dimensions: 30 mm (length) × 30 mm (breath) × 12 mm (height).

### 1.2. Microstructure of Rene 65

**Figure 3:**
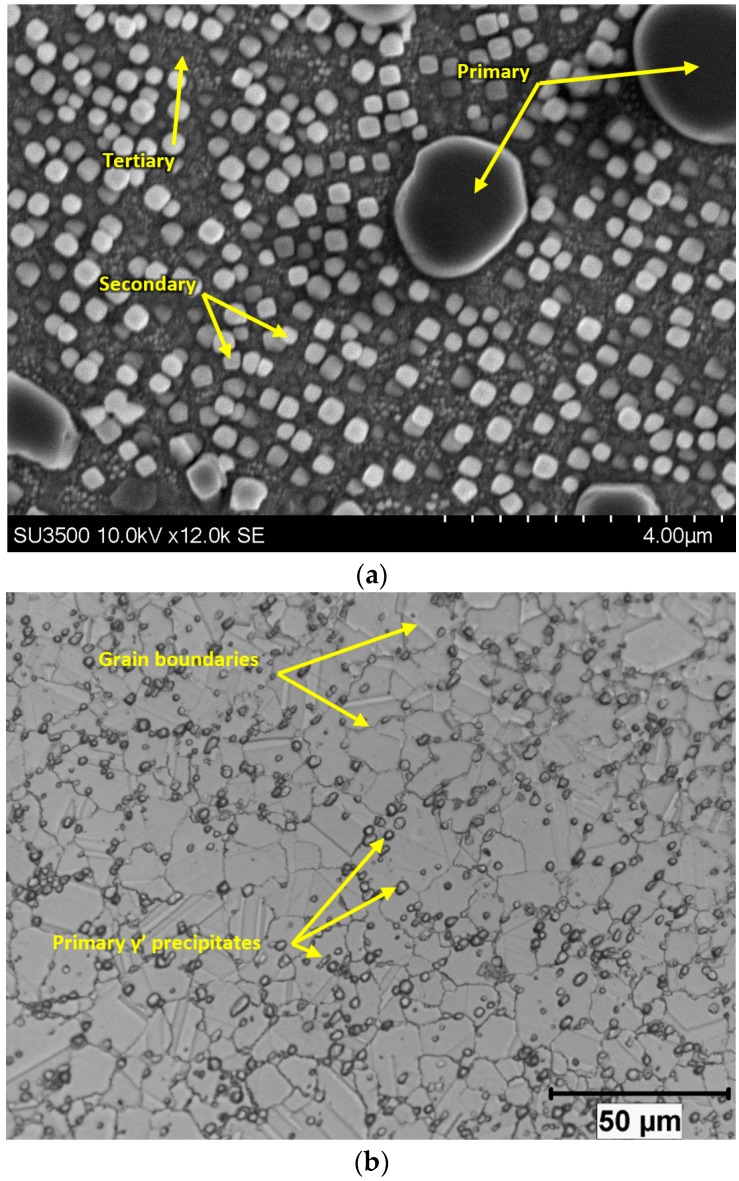
Microstructures of Rene 65 (**a**) Scanning electron microscopy (SEM) image showing the primary, secondary and tertiary γ′ precipitates, (**b**) Optical image of γ′ precipitates along grain boundaries.

The microstructure of Rene 65 consists of the Ni matrix, γ (gamma) and Ni3(Al,Ti) precipitates, γ′ (gamma prime). There are usually three different sizes of gamma prime: primary, secondary and tertiary, as can be seen from Figure 3a. The primary γ′ serves to pin grain boundaries and prevent grain coarsening and are thus usually found at grain boundaries (Figure 3b) while other precipitates served as a strengthening agent.

### 1.3. Strengthening of Ni Superalloys

Various techniques are used to improve strength of materials and they all have different effects on material properties. These techniques use either solid solution strengthening, strain hardening, precipitation hardening or grain size reduction. Solid solution strengthening by the addition of alloying elements is often used commercially. This addition provides strength and creep resistance by impeding crystallographic dislocations or by the precipitation of small coherent particles of intermetallic compounds [14]. Grain boundary strengthening, also known as the Hall-Petch effect, is a method of reinforcing material strength by modifying the average grain size [15]. It identifies the maintenance of grain boundaries and the reduction of dislocations as significant ways in improving material yield strength. Grain size can be controlled by an appropriate heat treatment through dynamic recrystallization (DRX) process in materials. The precipitation of phase elements could assist in impeding grain mobility through the pinning of grain boundaries. Precipitation hardening in Ni-based superalloys involves the precipitation of γ′ precipitates within the microstructure to improve material strength. This increase in strength could be due to an increase in the volume fraction of γ′ precipitates. At a given temperature, the volume fraction of γ′ precipitates correlates to the hardening elements present and affects the high temperature strength of the nickel alloy [14]. A study by Tancret, et al. [16] on the design of a dynamic recrystallization model in precipitation-hardened superalloys indicates the relationship between recrystallized grain sizes, temperature and strain rate. 

In the literature, Rene 65 is used in a subsolvus heat treated condition and as a result, a significant fraction of primary gamma prime is retained in the fully heat treated material [3], whereas the secondary and tertiary precipitates present within the grains contribute to the strength and creep resistance of the alloy [17]. Reference [3] found that the size distributions of the secondary and tertiary gamma prime were dependent on the cooling rate at the end of the heat treatment, with faster cooling rates leading to finer particles.

The mechanical properties of precipitation-hardened superalloys such as Rene 65 depend directly on the size and volume fraction of all the three types of precipitates [18]. For Rene 65 specifically, its hardness and yield strength are primarily dependent on the size and volume fraction of secondary and tertiary precipitates in the materials, as the fraction of primary precipitates and grain size are usually not modified in subsolvus heat treatment. Therefore, in this paper, various heat treatments were designed to alter the strength of the material by changing the size and the volume fraction of the secondary and tertiary precipitates.

### 1.4. Cutting Drill Materials

Carbides, high-speed steels, Cubic Boron Nitride (CBN) and Ceramics are frequently used tool materials for nickel-based superalloys [14]. The choice for selection for cutting tools requires the following properties: improved wear resistance, high strength and toughness, high temperature strength, improved thermal properties and chemical stability at elevated temperatures [14]. Carbide tooling is often used in Ni-based superalloys at operating ranges of 10–30 m/min when machining. These superalloys are capable of operating under interrupted cutting conditions and at increased feed rates [14]. However, due to the adverse effect of thermal instability, the tools cannot operate at elevated speeds [12]. The addition of a TiAlN coating layer on carbides improves the tools’ wear resistance and strength at slightly higher temperatures but this material is still susceptible to extreme wear conditions [19]. A study by Rosnan, et al. [20] on the drilling of nickel-titanium shows the advantage of using TiAlN-coated drills in terms of wear resistance improvement.

## 2. Experimental Setup

**Figure 4:**
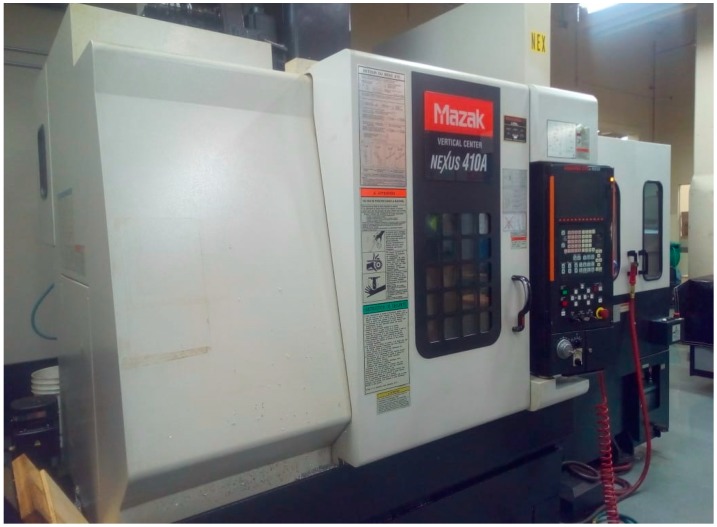
MAZAK NEXUS 410A CNC vertical milling machine.

Drilling tests were performed on a MAZAK NEXUS 410A CNC vertical milling machine (12000 RPM and 25HP) (Yamazaki Mazak Corporation, Oguchi, Japan) (Figure 4). The tests were conducted on three workpiece samples of varying hardness. The next sub-sections highlight the experimental components, such as the type tool, workpiece and measurements used in the study.

**Figure 5:**
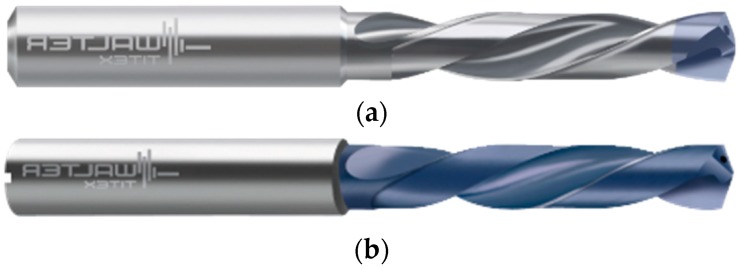
Drilling tools used. (**a**) Coating Grade: K30F (A3293TTP-3); (**b**) Coating Grade: WJ30RE (DC150-03).

Two distinct coated carbide drills were tested to identify the most favorable grade. These grades vary in coating applications along the flutes, flutes length, point type and geometry. Figure 5 indicates the two grades of drills used in the study. The grades vary fundamentally on the length of TiAlN coating deposited along the flutes and length of flute. Table 2 shows the properties of the drill bits. From Figure 5a, the application of coating is only applied at the tool tip; however, Figure 5b shows the coating all along the length of the flute. The length of the coating is believed to have an influence on the stress distribution on the tool tip and on the force generated during machining.

### 2.1. Experimental Design Plan

A detailed experimental analysis was carried out based on a standardized Box-Behnken design of experiment method of three input factors. This consisted of an array of 15 experiments used to estimate the effects of cutting speed, feed rate and material hardness on cutting forces and surface roughness. Table 3 shows the factors and their respective levels used in the experimental design.

**Figure 6:**
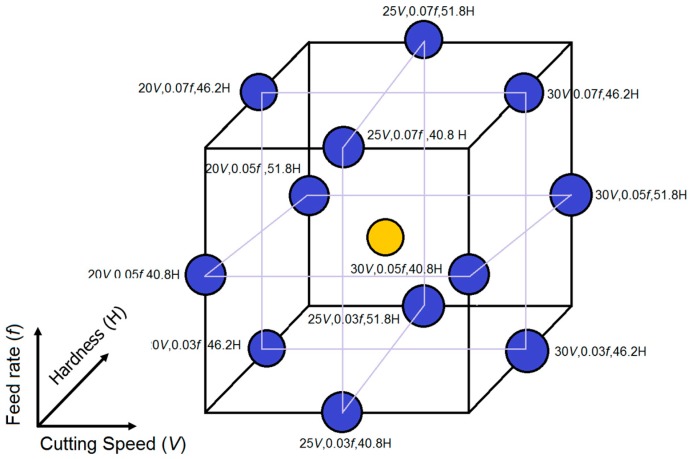
Box-Behnken experiment design.

The Box Behnken-based response surface method (RSM) offers the advantage of assessing a number of mid-range points in its design. This RSM method has been applied in the ultra-high precision machining of contact lenses [21] and in other machining techniques [22]. This methodology is best-suited for experimental tests with limitations on extreme ranges. As is evident in this study, there exists limitations in reaching some specific hardness values on Rene 65 samples by the heat treatment process. These extremes and functional ranges are delimited within the experimental plan and only plausible hardness values, which can be attained experimentally, are set in the experimental plan (Figure 6). To predict responses, outside experimental tested conditions, statistical models have been developed.

### 2.2. Heat Treatment

In this work, two of the above-mentioned as-deformed samples (AD) were subjected to subsolvus heat treatments. The main principle of the heat treatments was based on preventing excessive grain growth while altering the amount and size of precipitates. Both samples were first subsolvus solution-treated at 1095 °C and then one was water-quenched (WQ) and the other, air-cooled. The air-cooled sample was then subjected to a 6 h heat treatment at about 780 °C, followed by air-cooling (AC). After the heat treatment, three samples with different hardnesses were obtained: 41, 46 and 52 HRC for the WQ, AC and AD, respectively, measured by a Mitutoyo Rockwell Hardness tester, HR430. Details of the thermal treatment process for the samples are given in Table 4.

The microstructures of the three samples were characterized using a Hitachi SU3500 scanning electron microscope (SEM) (Hitachi Ltd., Tokyo, Japan). To reveal the γ′ under SEM conditions, electropolishing and electroetching were carried out.

### 2.3. Measurement of Response Factors

**Figure 7:**
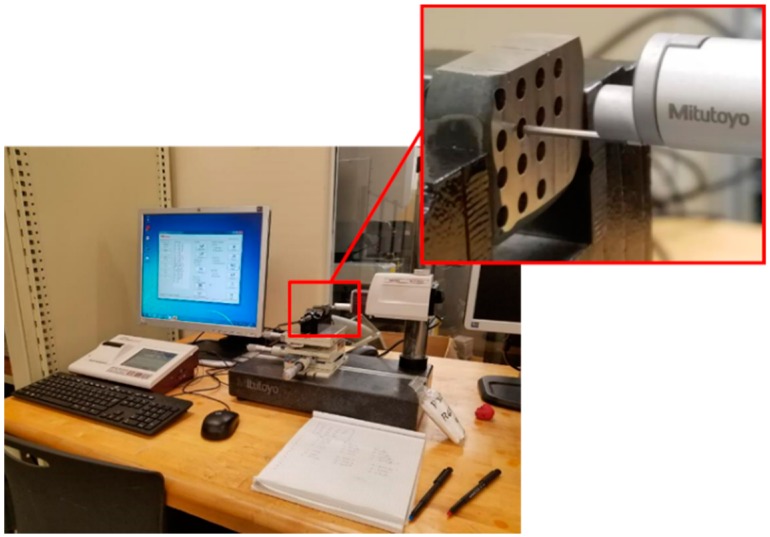
Mitutoyo profilometer and the setup for the roughness acquisitions.

The cutting force, surface roughness and cutting tool chip formation were observed and recorded in this study. The average surface roughness (Ra) was measured using the Mitutoyo SURFTEST SJ-410 roughness measuring instrument (Mitutoyo America Corporation, Illinois, United States). At least five measurements were carried out along the length of each drilled hole to ensure repeatability of results. The sample was then rotated to confirm the recorded roughness values across different orientations. Two sets of values depicting the lowest roughness obtained for each drilled hole and a mean value were recorded. Figure 7 shows the profilometer and the setup for the roughness acquisitions.

**Figure 8:**
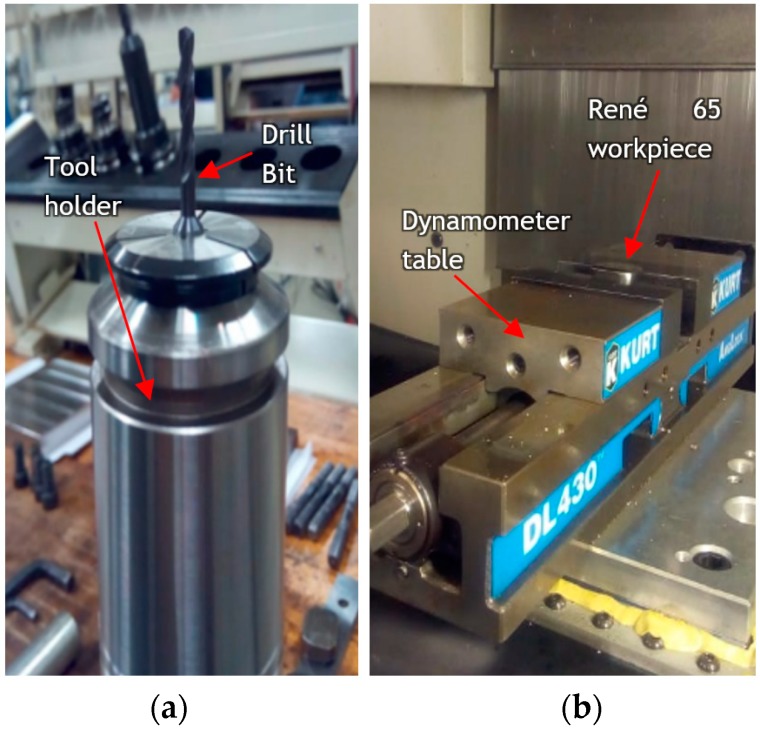
Tool setup: (**a**). Tool and tool holder (**b**). Mounted workpiece on force dynamometer.

Experimental cutting force acquisitions of three force components (the cutting force (Fx), the radial force (Fy) and the thrust force component (Fz)) were measured during drilling tests with a Kistler dynamometer type 9255B table installed in the Mazak CNC machine. The workpiece was mounted on a vice and set up on the dynamometer (Figure 8). The acquisitions were then collected by a data acquisition system, (DAQ) and amplified for further processing in the LabVIEW^®^ software (LabVIEW 2015, National Instrument, Austin, Texas, United States).

Because of the small size of the drill, a more stable tool holder with an increased length was used in the test. This was adapted to mitigate the effects of tool deviation from its center and prevent eventual tool breakdown.

## 3. Results and Discussions

### 3.1. Impact of Heat Treatment on Rene 65 Microstructures

**Figure 9:**
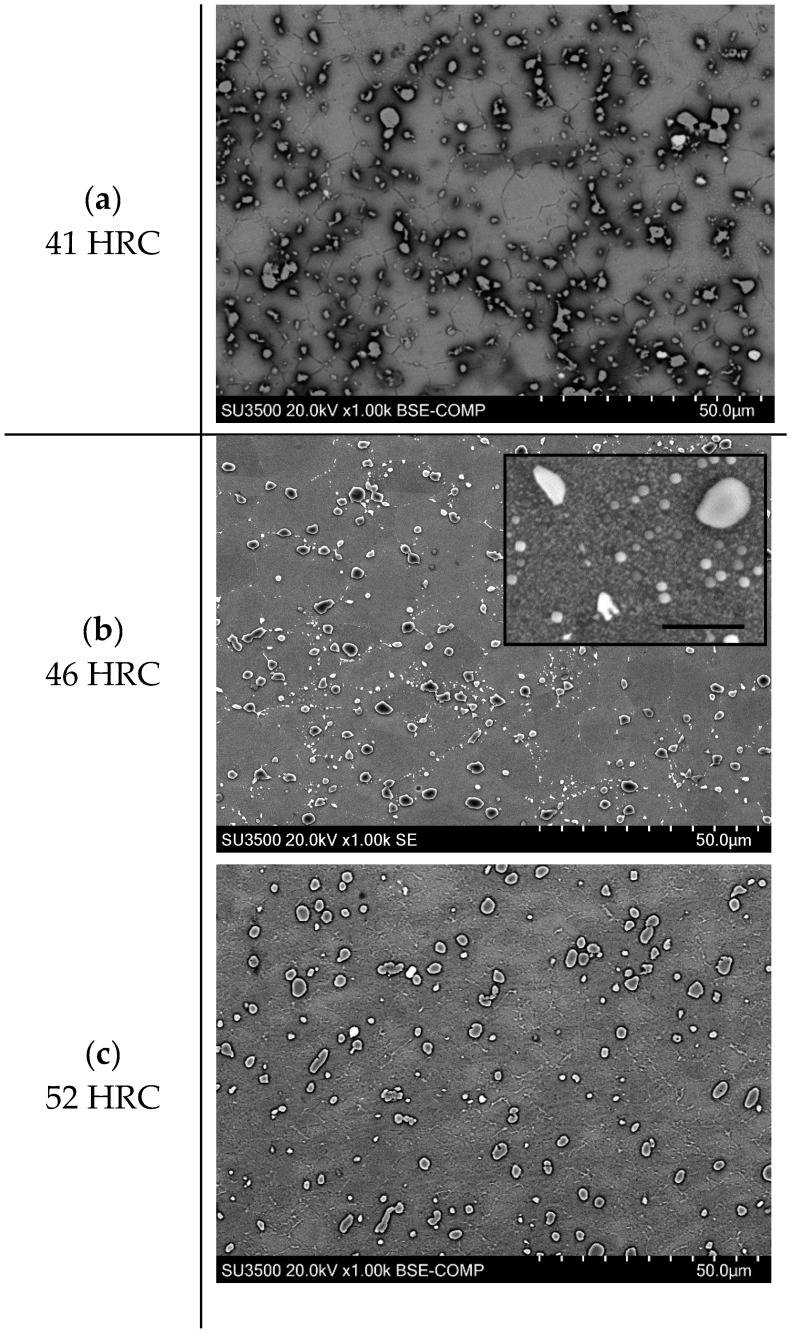
Rene 65 Microstructure at: (**a**). 41 HRC (**b**) 46 HRC (**c**) 52 HRC.

Figure 9 shows the microstructures of the three Rene 65 samples with different harnesses (note that the SEM images were obtained in secondary electron, SE and back-scattered electron (BSE) modes). It can be seen that the three specimens exhibit similar amounts of primary gamma prime precipitates, which are present at the grain boundaries. For the WQ sample, there are only few secondary precipitates within the grains, as shown in Figure 9a. The low amount of secondary precipitates and the absence of tertiary precipitates is due to the high cooling rate from water quenching, which suppressed the precipitation process. For the specimen with a 46 HRC hardness, a large amount of secondary and tertiary gamma prime precipitated within grains, as can be seen from Figure 9b and the higher-magnification inset. The emergence of secondary and tertiary gamma prime can be attributed to the relatively lower cooling rate and extra heat treatment as compared to the WQ sample. As for the sample with a 52 HRC hardness (Figure 9c), it is expected to have the highest amount of precipitates, which contribute to the highest hardness among the three specimens. However, the fine precipitates are not clearly revealed within the grains. Nevertheless, this paper only focuses on the influence of material hardness and the influence of microstructure on machinability will be covered in future work.

### 3.2. Effect of Drilling Tools

**Figure 10:**
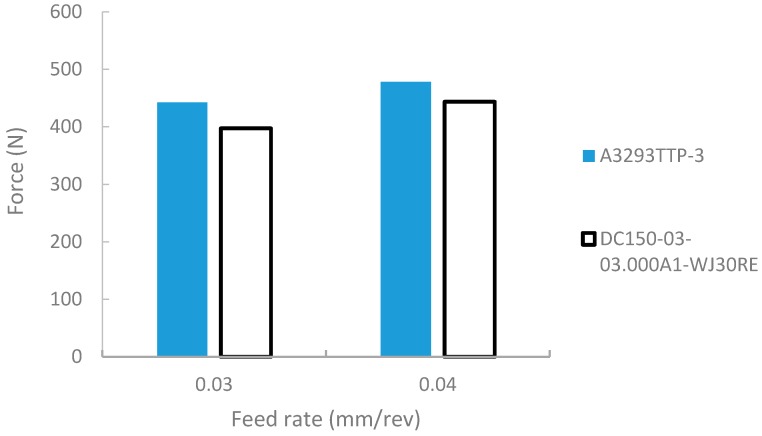
Effect of cutting tool coating grade on cutting force.

There are a couple of phenomena associated with the formation of wear during machining. Some of these are thermal softening, diffusion, abrasion and mechanical fatigue of the material [23]. Due to the occurrence of work hardening and poor thermal conductivity, nickel-based superalloys generate high cutting temperatures between the tool and the machined work surface [24]. These temperatures rise steadily during cutting due to the poor heat dissipation properties of the material, leading to rapid tool deterioration. Coating carbide tools greatly improves the tool resistance to wear. Various grades of tool coating exist in drilling, some of which include the application of TiAlN, TiN, AlCrN and multi-layer coating grades. Various criteria exist in specifying tool grades across manufacturers, which vary from modifications in edge preparations to the length and position of coat deposition along the length of the drill. In this study, the two grades, which vary on the application of TiAlN coating layer application along the flutes of the drill, show that “DC150-03” tools performed slightly better than “A3293TTP-3” tools, with reduced forces during drilling tests (Figure 10).

Although the heat generation in machining occurs at the distinct shear zones, it emanates from the tool tip point of contact and progresses with the chip along the tool-chip interface [25]. This heat generated on the shear plane, combined with friction, directly influences the temperature of the chip at the shear plane and the flow of heat at the rake face of the tool. Consequently, this further affects the shape of the predicted temperature profile along the cutting edges [26]. The impact of the tool choice is therefore influential beyond the tool coating depth, due to the heat transfer from chip evacuation. Therefore, it can be postulated that as the drill depth increases, the wear along the uncoated flutes increase the resistance to chip flow. Due to the low depth of cut of 9.5 mm used in our experiments, a negligible difference in forces was observed between the different coating lengths of the tools. However, an improved level of chip evacuation from its improved self-centering grade was observed on the tool A3293TTP-3. Therefore, “A3293” was selected as the experimental tool for successive tests in this study.

### 3.3. Surface Roughness

The surface roughness estimation is an important criterion in part performance for functionality. This criterion influences other factors, such as the wear rate, the friction coefficient, the corrosion resistance and the fatigue strength [27]. Conversely, it is influenced by a variety of factors, which are linked to the cutting tool (for example tool edge geometry, the rake angle), the workpiece (the material microstructure) and the cutting conditions (the feed rate, the cutting speed), among others. A statistical analysis of the lowest internal roughness values is depicted in the next section. The analysis of variance (ANOVA) enables the study of variability of the means of experimental observation as well as an examination of the significance of the factors [28]. Table 5 shows the ANOVA analysis for the lowest surface roughness.

**Figure 11:**
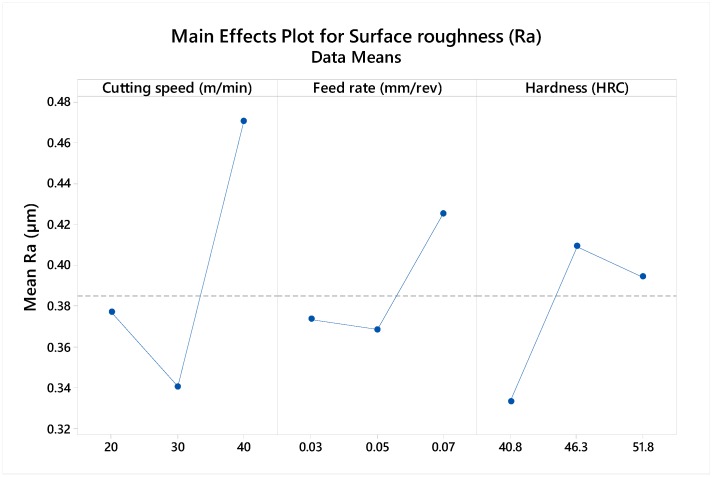
Main effect plot of Ra (μm) to cutting parameters.

From the table, the hardness and the feed rate are insignificant, with a P-value of 0.380 and 0.38, respectively. However, the P-value of the cutting speed, the square of the feed rate and the interaction of the speed and hardness are identified as significant terms within a 95% confidence level. The main effect plot in Figure 11 further highlights the influence individual factors have on the surface response. From the plot, the cutting speed is identified as the most significant term to surface finish, with a significantly higher mean at increased speed value. A lower effect is observed from the feed rate and the hardness.

**Figure 12:**
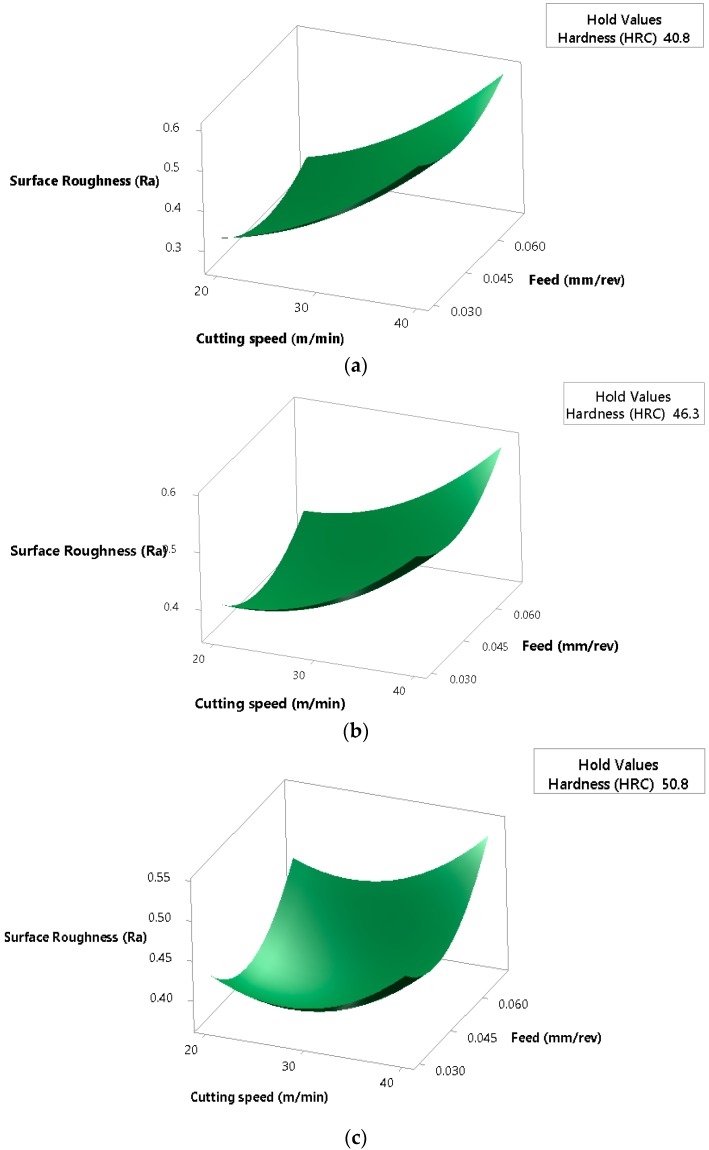
Surface plots of roughness (Ra) versus cutting speed and feed rate at hardness values of: (**a**) 41 HRC (**b**) 46 HRC and (**c**) 52 HRC.

The surface plots of the results reveal that in samples with lower hardnesses, there is a correlation between rising cutting speed and feed rates with rougher surfaces (Figure 12a,b). Therefore, improved surfaces at hardness levels of 41 and 46 HRC are obtained at low feed and cutting speeds. However, at increased hardnesses, optimal surface conditions are found at average speed and feed values, with increased roughness seen at lower and higher cutting conditions (Figure 12c).

This is believed to stem from the effect of the recoil of the harder chips on the internal surface of the drilled holes during evacuation.

### 3.4. Cutting Force Analysis

From the cutting forces measured during drilling, only the thrust force (*F_z_*) was selected to study the influence of cutting parameters in the statistical analysis. This force component is responsible for the penetration of the tool into the workpiece and is the most significant force in drilling operations, while the cutting force (*F_x_*) represents the force in the direction of cutting speed. These two forces are higher in magnitude than the radial force component (*F_y_*), which act on each lip of the drill towards its center and often counterbalances itself to generate a value close to zero.

#### 3.4.1. Main Effect Plots of Cutting Forces

**Figure 13:**
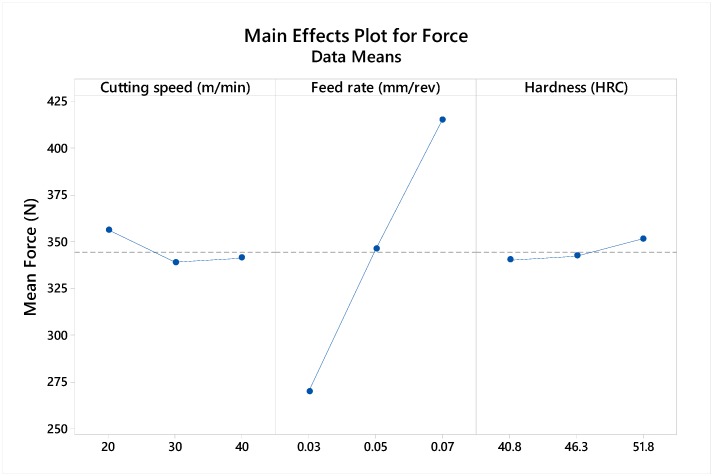
Main effect plot of cutting force to cutting parameters.

A cutting force analysis of the drilling process revealed a high correlation of feed rate to generated forces. The influence of other parameters, such as the workpiece hardness and the cutting feed, has a negligible effect on the generated forces. As a result, an increase in feed rate generated a corresponding rise in forces (Figure 13). This occurrence linked to feed is assumed to be due to the increased chip thickness, as well as the force requirement during chip shearing.

The development of an adequate regression model, which captures the influence of cutting parameters, could be used for predictions beyond the range of experiments studied. The cutting forces in high feed machining could thus be estimated. The next sub-section evaluates various modeling approaches to establish forces at higher feed rates.

#### 3.4.2. Analysis of Cutting Force Prediction Models

Various modelling approaches were tested in this section to identify the differences in prediction. In this study, a linear model, the single parameter model, full quadratic and power form model have been developed. A Pearson test with a significance level α = 0.05 (95 percentile accuracy) was used to identify significant terms.

(a) Linear model: Table 6 shows the ANOVA table for the linear solution. From the table, the model developed recognized as significant to the data, also indicating the significance of the feed with a Pearson test value of 0.000. The model summary indicates a 96.17% r-square and 92.13% r-square predicted value. From the lack of fit test for the model, we cannot conclude that there is a lack of fit for the model with a 0.069 P-value. The model equation is shown in Equation (1).
(b) Full quadratic equation with interactions: The ANOVA of the quadratic solution is shown in Table 7. The quadratic expression also shows high significance of the quadratic model to the captured problem with no improved significance from interactions and squares. The feed rate also possesses a 100 percent significance in p-test estimation. However, the summary of this model indicates a higher R-square value of 98.74% and 80.68% R-square predicted. The regression equation of the quadratic representation is seen in Equation (2).
(c) Significant factor equation: The ANOVA table, which only includes significant factors, is shown in Table 8. This table indicates the feed rate as being the only significant factor to force prediction. Through a stepwise regression with an alpha value of 0.1 all non-significant factors have been removed during model formulation. A slightly lower R-square value of 94.56% is obtained with 94.15% improved adjusted and 92.80% predicted r-square values. The regression equation based only on the feed rate is shown in Equation (3).
(d) Power form equation: In power or exponential regression, the function is a polynomial equation of the form *y* = *ax^b^*. This form of equation allows us to model a problem whose factors do not increases in a non-linear fashion. Power regression is one in which the response directly relates to the input factor raised to a given power. It can be obtained by first taking the log of both factors and response values and then performing a least square regression on the transformed data and finally, an inverse transformation to ascertain that the resulting power function captures the trend of the data. A lower but still acceptable, R-square value of 93.86% with a 91.81% r-square predicted value is obtained. The inverse transformation of the regression function of Equation (4) is shown in Equation (5).

Regression Equation:

(1) Force (N)=137.6−0.762V+3635f+1.029H

where *V* is the cutting speed (m/min); *f* is the feed rate (mm/min); *H* is the hardness of the material.

Regression Equation

(2) $$Force\;\left(N\right)=657-16.42V+6804f-15.0H+0.0999V^{2}-5613f^{2}+0.131H^{2}-6.6Vf+0.2159VH-52.0fH$$

Regression Equation

(3) Force (N)=162.4+3635f

Regression Equation

(4) lnForce=5.2889+10.783f

(5) $$Force\;\left(N\right)=198.125\times f^{10.783}$$

#### 3.4.3. Graphical Comparison of Model Predictions

**Figure 14:**
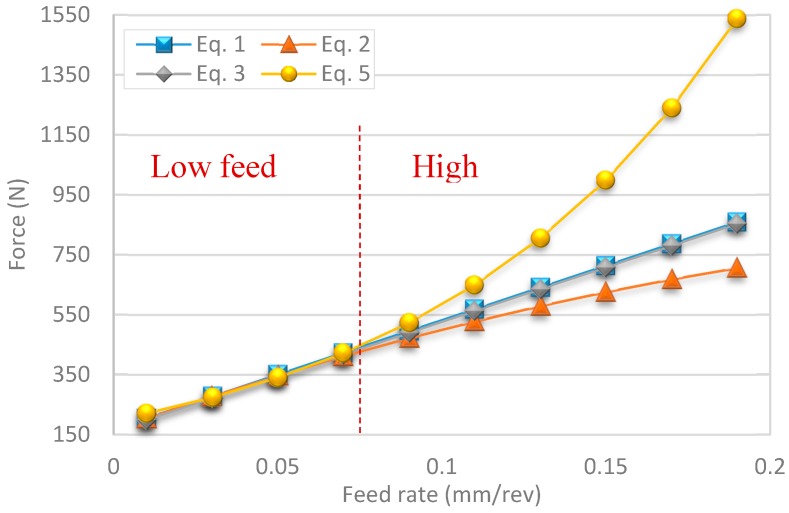
Comparison of modeling technique on the effect of change in feed rate on cutting forces.

From Figure 14, a side-by-side comparison of the different experimental predictions using the different model solutions is shown. A close performance can be observed from each of these models with minor deviations at lower feeds. Power form equations had the highest predicted force values over experimental test conditions, while the quadratic model had the lowest in its predictions. The average values for force across models were reflected mostly from the feed-only model equation.

A review of predictions beyond experimental test parameters shows higher variation trends. The main advantage of high feed machining lies in the increased productivity it provides in roughing operations. Some experimental studies in the literature also employ similar feed rates ranging from 0.05 to 0.1 mm/rev in the drilling of γ-TiAl intermetallic alloys [29] and from 0.05 to 0.15 mm/rev in the drilling of Inconel 825 [30]. In Figure 14, an exponential increase in force experienced is predicted in the power form model equation. Linear and feed-only approaches present reciprocal overlapping results with a small error margin in differences in prediction. However, the quadratic model, which has the highest fitting to the solution, with an r-square of 98.74%, shows a slight negative bend in forces obtained during high feed machining.

### 3.5. Wear and Lubrication in Machining Rene 65

**Figure 15:**
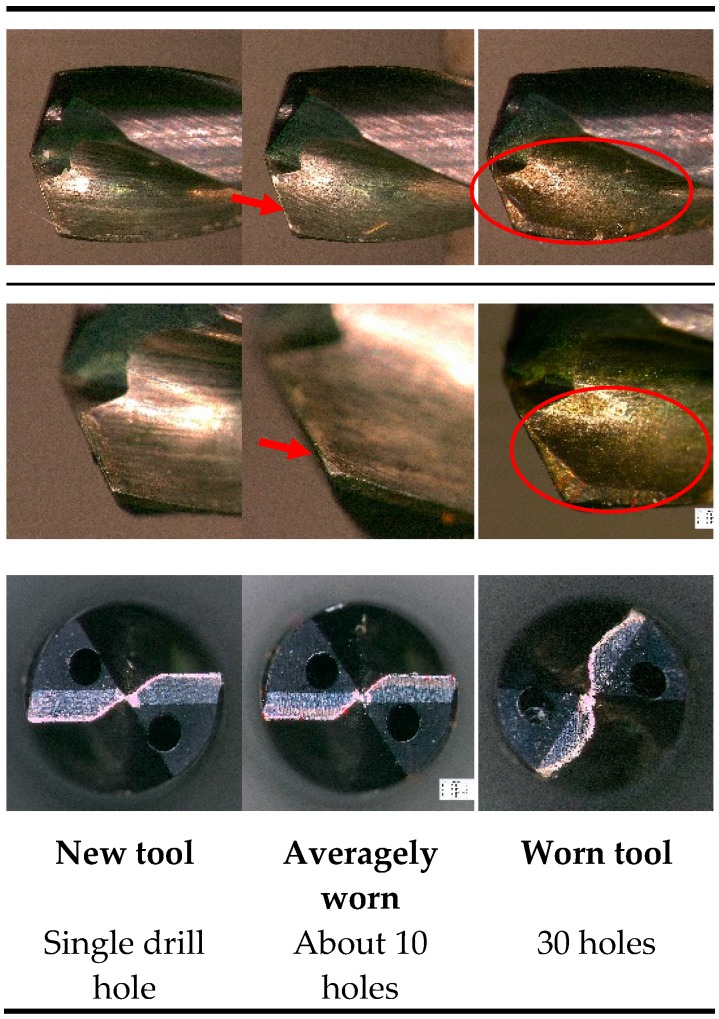
Discoloration of cutting tool tip due to high generated temperatures at the edges.

Wear formation observed during the drilling tests were mostly found on the flank wear. Due to the high abrasive forces experienced on the tool cutting edges with nickel superalloys, an erosion of the cutting flank was observed. Figure 15 shows the rapid wear progression on the flank face of the tool.

The cutting fluid plays a vital role in machining these high temperature superalloys. Coolant is often applied in cutting experiments to reduce cutting forces and provide lubrication between the tool and the workpiece in a bid to lower the cutting temperatures due to excessive abrasive forces. 

**Figure 16:**
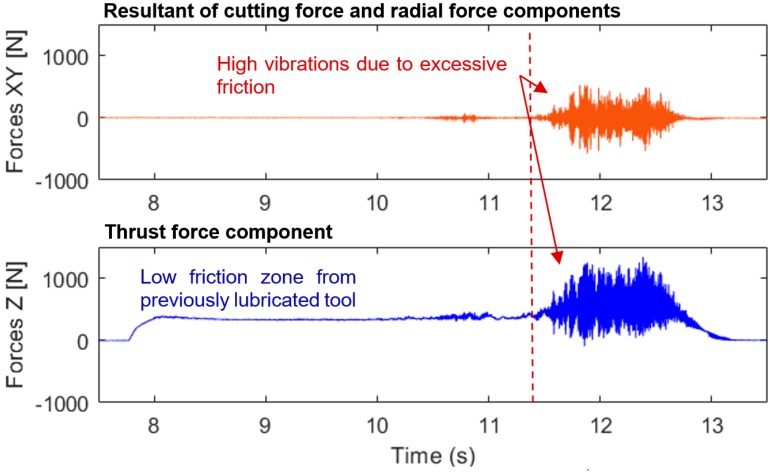
Cutting forces profiles during dry drilling test of Rene 65.

In drilling operations, temperatures easily rise in holes thanks to reduced dissipation, which therefore makes the use of coolants indispensable. A dry experimental test revealed a rapid rise in cutting forces from chips clogged within the drilled hole due to poor chip evacuation. This condition led to the eventual rupture of the tool with forces exceeding 1500 N (Figure 16).

The coolant application mode used to dissipate temperature also plays an important role. Coolant-through, which was later used for all experimental tests, showed an improved drilling performance in lubrication and force reduction over flooding during experimental trials.

### 3.6. Cutting Chip Analysis

The shape of tool chips is influenced by both the orbiting drilling process and the temperature gradient experienced on opposing faces of the chips. This gradient further enhances the chip curls and as a result, affects tool wear. Cutting parameters have also been known to affect the chip thickness, texture, color and edges. 

**Figure 17:**
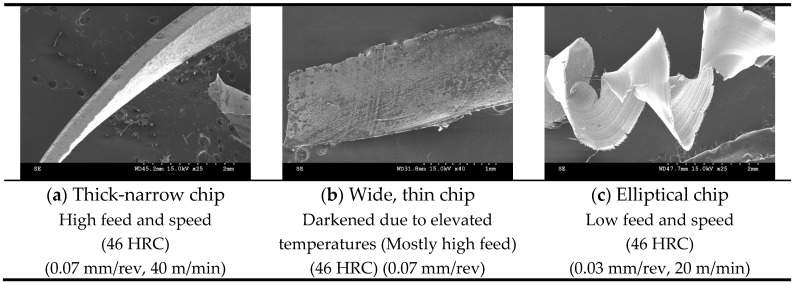
Different types of chip morphology observed. (**a**) Thick-narrow chip; (**b**) Wide, thin chip; (**c**) Elliptical chip.

Effective chip segmentation was achieved while drilling Rene 65 with coated coolant-through carbide tools. These chips, formed following severe plastic deformation in the primary shear zone, were therefore flushed out of the drilled hole with high internal pressure coolant pressure. Although few continuous chips were produced in this process, at low feed and low speed on the 46 HRC sample, an elliptical chip formation was observed (Figure 17c). This was not commonly seen in the study with most of the chips in other conditions segmented. It is evident that chip fragmentation was beneficial in promoting heat dissipation during cutting. 

Three different types of cutting chip morphologies have been identified in this study (Figure 17). As shown in the figure, thick narrow, flat and thin and elliptical chip forms were recovered from experimental tests. Color differences were seen, with darker tool chips observed mostly at higher feeds, which shows how feed rates affect temperatures during drilling.

**Figure 18:**
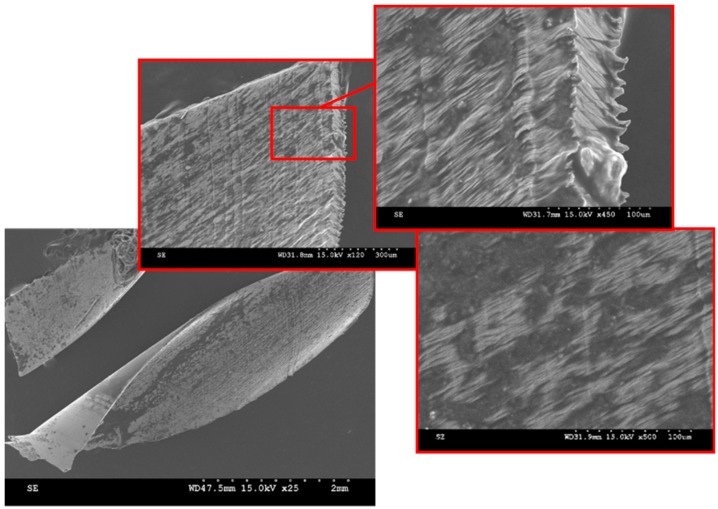
Surface microstructure and chip color due to elevated temperature in the drilled holes.

Contingent to the materials’ thermal conductivity, about 80 percent of heat generated during machining is transferred by the separating chip [31]. During a cutting operation, the increase in temperature, combined with the oxidative stability of the coolant, creates an oxide film on the chip surface. Darker tool chip colors experienced in the cutting of steels have been associated with temperatures above 860 °C at the cutting tool tip [31]. In their study on chip morphology, transformation and oxidation of hardened H13 tool steel, Zhang and Guo [32] associated the change in chip color observed with temperatures exceeding 1478 °C, the temperature at which water decomposes into its constituent elements. Similarly, the tool can also serve as an indicator of temperatures attained during cutting. A study by Yeo and Ong [33] highlights the influence of the thermal conductivity of coatings on heat generated at the interface between the tool and workpiece and consequently, the chip color. For the selected tool, aluminum titanium nitride (TiAlN), physical vapor deposition (PVD) coatings have a thermal service temperature of 788 °C, prior to indication of oxidation along the tool flutes [34]. This temperature is thus presumed to be within the temperature range of operation in the drilling tests of Rene 65 in this study. Figure 18 shows the discolored chip surfaces due to high temperature. High tool tip temperatures cause excessive tool wear, which reduce productivity. These temperatures can range from 1100 °C to 1300 °C and in turn, may cause severe wear and plastic deformation of the cutting tool edge [6].

**Figure 19:**
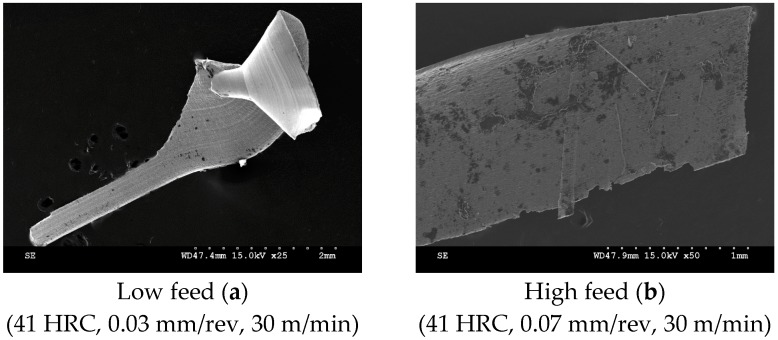
Cutting tool chips of 41 HRC Rene 65 samples at: (**a**) low feed, (**b**) high feed.

During the initial tool condition, the temperature plays an essential role in the chip shearing process. Successive influence comes from initiation of wear and the frictional outcome with the chip. In 41 HRC samples, short, thin and blackened chips were often identified at higher feed rates. On the other hand, at lower feed rates, a form of tail-like chip formation was initiated (Figure 19).

**Figure 20:**
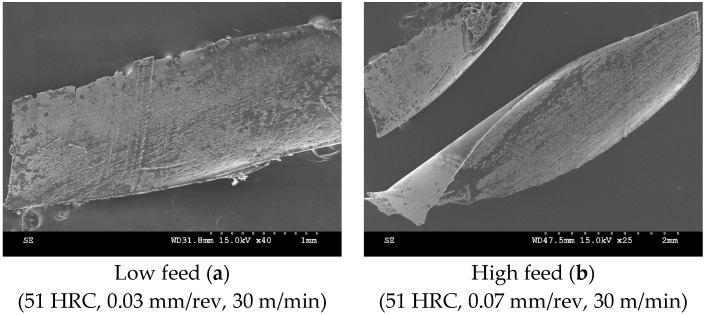
Cutting tool chips of 51 HRC Rene 65 samples at: (**a**) low feed, (**b**) high feed.

At 51 HRC, both high and low feed rates were indicative of increased temperatures in cutting, with the generation of darkened cutting chips (Figure 20).

**Figure 21:**
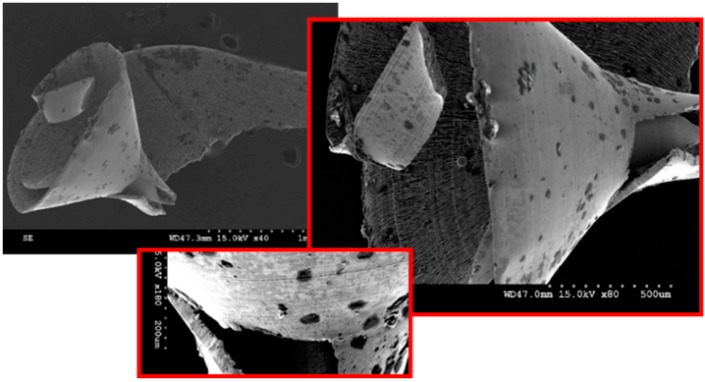
Cutting tool chips of the 51 HRC samples (0.05 mm/rev, 40 m/min).

However, the increase in cutting speeds in these harder samples promoted the initiation of a spiral chip. This was due to the increased temperatures, which resulted in a lower flow stress in the secondary shear zone and an improved frictional state between the tool-chip interfaces. However, due to the hard nature and lower ductility of the chips, tearing was frequently observed on them. (Figure 21).

**Figure 22:**
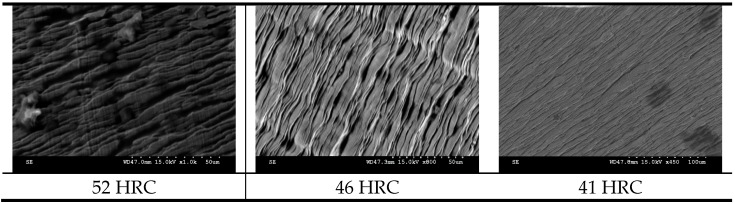
Microstructure of chips obtained at tested hardnesses.

The microstructures of the three samples revealed an increased residual stress on harder samples, with more pronounced lamella from frequent rupture during material shearing (Figure 22).

## 4. Conclusions

With a view to establishing the machinability of Rene 65, this study performed drilling tests on three Rene 65 samples with distinct microstructures and hardnesses. The results of the influence of the microstructure (with varying hardnesses) and machinability indicators (surface roughness, force and chip formation) on machinability are enumerated below:

- In general, the 41 HRC samples presented an improved machinability with reduced cutting forces and surface roughness. However, the range of hardnesses tested did not demonstrate a significant influence on the generation of cutting forces in drilling operations.
- The machinability of nickel-based alloys can be affected by the grade of the coating applied on the tool.
- The cutting feed rate was identified as the most significant parameter in the generation of cutting forces, while the cutting speed was more significant in the generation of surface roughness.
- The cutting parameters had a significant effect on the morphology of the chip and on the segmentation observed in harder samples.

## Figures and Tables

**Table 1:** Ni-based alloy materials with composition (wt.%) adapted from [2,13].

Materials ID	Rene65	AD730	In.718	Wasp	PER72
	Chemical Composition				
Nickel (Ni)	Bal.	Bal.	Bal.	Bal.	Bal.
Iron (Fe)	1	4.00	18.00	-	0.138
Cobalt (Co)	13	8.50	-	13.25	14.5
Chrome (Cr)	16	15.70	18.1	19.4	16.06
Molybdenum (Mo)	4	3.1	2.9	4.25	2.88
Tungsten (W)	4	2.7	-	-	1.21
Aluminum (Al)	2.1	2.25	0.45	1.3	2.57
Titanium (Ti)	3.7	3.4	1.00	3	5.07
Niobium (Nb)	0.7	1.1	5.4	-	-
Boron (B)	0.016	0.01	-	0.006	0.016
Carbon (C)	-	0.015	-	-	0.017
Zirconium	0.05	0.03	-	0.05	-

**Table 2:** Tested materials’ composition and hardness.

**Drill Bit Material**	**Solid Carbide**	**Number of Flutes**	**2**
Drill Bit Point Angle	140 Degrees	Diameter Size	3 mm
Drill Bit Finish	TiNAl	Coolant Through	Yes
**A3293**		**DC150**	
Flute Length (A3293)	26 mm	Flute Length (DC150)	20 mm
Point Type (A3293)	Self-centering	Point Type (DC150)	Split point
Grade	K30F	Grade	WJ30RE
Extreme Inox—Extremely high performance in stainless steels		Walter Titex DC15—Versatile and highly wear resistant	

**Table 3:** Factors and levels for testing Rene 65 drilling tests.

Factor	Level 1	Level 2	Level 3
Cutting Speed (m/min)	20	30	40
Feed Rate (mm/rev)	0.03	0.05	0.07
Hardness (HRC)	41	46	52

**Table 4:** Heat treatment processes for the distinct workpiece samples.

Hardness of Workpiece Material	Heat Treatment Process		
	Temperature	Time	Cooling
41 HRC	1095 °C	30 min	Water quench
46 HRC	1095 °C	30 min	Air cool
	788 °C	4 h	
	788 °C	2 h	
52 HRC	No treatment		

**Table 5:** Analysis of variance (ANOVA) for the lowest surface roughness.

Source	DF	ADJ SS	ADJ MS	F	P
Model	9	0.085467	0.009496	5.93	0.032
LINEAR	3	0.048963	0.016321	10.20	0.014
A: Speed (m/min)	1	0.047278	0.047278	29.54	0.003
B: Feed (mm/tooth)	1	0.001485	0.001485	0.93	0.380
C: Hardness (HRC)	1	0.000200	0.000200	0.12	0.738
Square	3	0.020869	0.006956	4.35	0.074
Speed × Speed	1	0.006500	0.006500	4.06	0.100
Feed × Feed	1	0.010950	0.010950	6.84	0.047
Hardness × Hardness	1	0.002955	0.002955	1.85	0.232
2-way Interaction	3	0.015635	0.005212	3.26	0.118
Speed × Feed	1	0.000210	0.000210	0.13	0.732
Speed × Hardness	1	0.014641	0.014641	9.15	0.029
Feed × Hardness	1	0.000784	0.000784	0.49	0.515
Error	5	0.008002	0.001600		
Lack-of-fit	3	0.003081	0.001027	0.42	0.761
Pure Error	2	0.004921	0.002460		
Total	14	0.093469			

Degree of freedom (DF), Adjusted Sum of squares (ADJ SS), Adjusted mean square (ADJ MS), F-test (F), Pearson value (P).

**Table 6:** ANOVA for linear model solution.

Source	DF	ADJ SS	ADJ MS	F	P
Model	3	43,011.6	14,337.2	92.17	0.000
LINEAR	3	43,011.6	14,337.2	92.17	0.000
A: Speed (m/min)	1	464.0	464.0	2.98	0.112
B: Feed (mm/tooth)	1	42,291.4	42,291.4	271.87	0.000
C: Hardness (HRC)	1	256.1	256.1	1.65	0.226
Error	11	1711.1	155.6		
Lack-of-Fit	9	1684.0	187.1	13.80	0.069
Pure Error	2	27.1	13.6		
Total	14	44,722.8			

**Table 7:** ANOVA for full quadratic equation.

Source	DF	ADJ SS	ADJ MS	F	P
Model	9	44,159.3	4906.6	43.54	0.000
LINEAR	3	43,011.6	14,337.2	127.23	0.000
A: Speed (m/min)	1	464.0	464.6	4.12	0.098
B: Feed (mm/tooth)	1	42,291.4	42,294.4	375.30	0.000
C: Hardness (HRC)	1	256.1	256.1	2.27	0.192
Square	3	445.6	148.5	1.32	0.366
Speed × Speed	1	368.6	368.6	3.27	0.130
Feed × Feed	1	18.6	18.6	0.17	0.701
Hardness × Hardness	1	57.8	57.8	0.51	0.506
2-way Interaction	3	702.2	234.1	2.08	0.222
Speed × Feed	1	7.1	7.1	0.06	0.812
Speed × Hardness	1	564.1	564.1	5.01	0.075
Feed × Hardness	1	131.0	131.0	1.16	0.330
Error	5	563.4	112.7		
Lack-of-Fit	3	536.3	178.8	13.18	0.071
Pure Error	2	27.1	13.6		
Total	14	44,722.8			

**Table 8:** ANOVA for with stepwise regression of alpha 0.1.

Source	DF	ADJ SS	ADJ MS	F	P
Regression	1	42,291.4	42,291.4	226.13	0.000
Feed (mm/rev)	1	42,291.4	42,291.4	226.13	0.000
Error	13	2431.3	187.0		
Lack-of-Fit	11	2404.2	218.6	16.12	0.060
Pure Error	2	27.1	13.6		
Total	14	44,722.8			
